# Association between Dysgeusia and Atherosclerotic Cardiovascular Disease: A Cross-Sectional Study With Propensity Score-Matched Analyses

**DOI:** 10.3290/j.ohpd.c_2528

**Published:** 2026-02-24

**Authors:** Peng Zhou, Xiaofeng Zhou, Su Tang, Defeng Liang, Donglei Wu

**Affiliations:** a Peng Zhou Dentist, Department of Stomatology, Guangdong Women and Children Hospital, Guangzhou, Guangdong, China. Study design, conducting the study, analysing the data, writing and reviewing the manuscript.; b Xiaofeng Zhou Dentist, Yichang Central People’s Hospital, China Three Gorges University, Yichang, Hubei, China. Study design, conducting the study, analysing the data, writing and reviewing the manuscript.; c Su Tang Dentist, Department of Stomatology, Shenzhen People’s Hospital, Shenzhen, China. Study design, conducting the study, analysing the data, writing and reviewing the manuscript.; d Defeng Liang Dentist, Department of Stomatology, Shenzhen Baoan Women’s and Children’s Hospital, Jinan University, Shenzhen, China. Study design, conducting the study, analysing the data, writing and reviewing the manuscript.; e Donglei Wu Dentist, Department of Stomatology, Shenzhen People’s Hospital, Shenzhen, China. Study design, conducting the study, analysing the data, writing and reviewing the manuscript.

**Keywords:** atherosclerotic cardiovascular disease, dietary behaviour, dysgeusia, inflammation, propensity score matching

## Abstract

**Purpose:**

Dysgeusia impairs quality of life and nutrition, and may be linked to cardiovascular disease risk, but its direct association with atherosclerotic cardiovascular disease (ASCVD) remains understudied. This study aimed to investigate the independent association between dysgeusia and ASCVD.

**Methods and Materials:**

We conducted a cross-sectional analysis using a nationally representative population sample. The association between dysgeusia and ASCVD was evaluated using multivariable logistic regression, adjusting for age, sex, race/ethnicity, education, body mass index, smoking, alcohol consumption, diet quality, hypertension, hyperlipidemia, periodontitis, diabetes mellitus, Parkinson’s disease, and depression. Propensity score matching (PSM) was employed to further control for confounding and to validate the findings.

**Results:**

In the unadjusted model, dysgeusia was significantly associated with higher odds of ASCVD (odds ratio [OR] = 1.88, 95% confidence interval [CI]: 1.39–2.55; P < 0.01). After full adjustment, the association remained significant (OR = 1.46, 95% CI: 1.01–2.13; P = 0.05). PSM analysis further confirmed this relationship, showing that individuals with dysgeusia had 87% greater odds of ASCVD compared to matched controls (OR = 1.87, 95% CI: 1.08–3.23; P = 0.03).

**Conclusion:**

Dysgeusia is independently associated with increased odds of ASCVD, even after comprehensive adjustment and PSM. Dysgeusia may serve as a novel and clinically relevant potential indicator for ASCVD, warranting further investigation in prospective studies.

Atherosclerotic cardiovascular disease (ASCVD) remains the leading cause of morbidity and mortality globally, accounting for over 19 million deaths annually.^40^ Although traditional risk factors such as hypertension, dyslipidemia, and diabetes mellitus have been extensively characterised,^9^ emerging evidence suggests potential associations between sensory dysfunction and cardiovascular pathophysiology.^20^

Dysgeusia (taste disorders) affects approximately 0.6–20% of adults worldwide,^20^ with higher prevalence observed among older adults and individuals with chronic diseases.^4,32,36^ Recent preclinical studies indicate that transient receptor potential (TRP) channels, which are crucial for taste perception,^15,21^ may also modulate vascular endothelial function.^19^ Additionally, clinical observations have reported altered dietary patterns among individuals with dysgeusia, which may contribute to an increased risk of ASCVD.^18,36^ Notably, zinc deficiency – a common aetiological factor in taste dysfunction^1^ – has been independently associated with both endothelial dysfunction^43^ and arterial stiffness.^1^ Collectively, these mechanistic and clinical insights support a plausible biological link between dysgeusia and ASCVD, leading us to hypothesise a direct association between the two conditions. However, current epidemiological evidence primarily focuses on combined smell and taste disorders in relation to cardiovascular disease (CVD),^20^ with scant investigation into isolated taste impairment. To address this knowledge gap, this cross-sectional study aims to systematically investigate the association between self-reported dysgeusia and ASCVD, using nationally representative data from the National Health and Nutrition Examination Survey (NHANES).

## METHODS AND MATERIALS

### Data Source and Study Population

This cross-sectional study was based on data from the NHANES 2011–2014, a nationally representative survey designed to assess the health and nutritional status of the civilian, non-institutionalised US population. The NHANES protocol was reviewed and approved by the National Center for Health Statistics (NCHS) Institutional Review Board, and written informed consent was obtained from all participants. All study procedures were conducted in accordance with the Strengthening the Reporting of Observational Studies in Epidemiology (STROBE) guidelines and the ethical principles outlined in the latest version of the Declaration of Helsinki. Collected variables included demographic characteristics (age, sex, ethnicity, educational level, body mass index [BMI]), lifestyle factors (smoking, alcohol consumption, Healthy Eating Index-2015 [HEI]), systemic disease history (hypertension, diabetes mellitus, hyperlipidemia, Parkinson’s disease, depression and periodontitis), and dysgeusia assessments relevant to ASCVD risk.

### Definition of ASCVD

The primary outcome was ASCVD, defined according to the 2013 American College of Cardiology/American Heart Association (ACC/AHA) Guideline on the Treatment of Blood Cholesterol to Reduce Atherosclerotic Cardiovascular Risk in Adults.^35^ ASCVD was identified based on a self-reported history of coronary heart disease, angina, myocardial infarction, or stroke, as determined from the NHANES medical conditions questionnaire.

### Definition of Dysgeusia

Dysgeusia was assessed using the standardised Taste & Smell Questionnaire from NHANES,^28^ which collects comprehensive self-reported data on gustatory and olfactory function. Eligible participants included adults aged 40 years or older. Individuals were classified as having dysgeusia if they reported any of the following during the preceding 12 months: taste-related problems, a diminished ability to perceive the flavour of foods, or persistent alterations in the perception of basic taste qualities (salt, sour, sweet, or bitter).

### Covariates

Covariates were selected based on established risk factors for ASCVD and were extracted from the NHANES 2011–2014 database. Demographic information, including age, sex, race/ethnicity (categorised as non-Hispanic White, non-Hispanic Black, Mexican American, or Other), and education level (< 9th grade, 9th–12th grade, or >12th grade), was collected. Furthermore, BMI was recorded as an anthropometric indicator, while lifestyle factors such as smoking status (current smoker or non-smoker), alcohol consumption (current drinker or non-drinker), and dietary quality – measured by the HEI – were included to capture relevant behaviours. Medical history variables comprised hypertension, diabetes mellitus, hyperlipidemia, Parkinson’s disease, depression, and periodontitis, each defined according to established clinical or laboratory criteria, or medication use when appropriate. For instance, hypertension was identified through measured systolic/diastolic blood pressure ≥ 140/90 mmHg, self-reported diagnosis, or antihypertensive medication use; diabetes was recognised by self-reported diagnosis, HbA1c ≥ 6.5%, fasting plasma glucose ≥ 7.0 mmol/L, oral glucose tolerance test ≥ 11.1 mmol/L, or use of antidiabetic drugs. Hyperlipidemia was indicated by any of the following: total cholesterol ≥ 200 mg/dL, triglycerides ≥ 150 mg/dL, LDL-C ≥ 130 mg/dL, low HDL-C (≤ 50 mg/dL in females or ≤ 40 mg/dL in males), or the use of cholesterol-lowering medications. Periodontitis was classified according to the CDC/AAP 2012 guideline, while Parkinson’s disease was identified through the prescription of antiparkinsonian agents. Depression severity was evaluated using the nine-item Patient Health Questionnaire (PHQ-9), with clinically significant depression defined by a total score of 10 or higher. Additionally, prescription medication use in the 30 days prior to the survey was documented for all participants, allowing for further adjustment of potential confounders in the analyses.

### Statistical Analysis

All statistical analyses accounted for the complex sampling design of NHANES by applying appropriate survey weighting throughout both descriptive and inferential procedures. Continuous variables were summarised as mean ± standard deviation (SD) if normally distributed, or as median with interquartile range (IQR) when distributions were skewed. Categorical variables were reported as frequencies and percentages. Differences in non-normally distributed continuous variables – such as age, BMI, and HEI scores – between dysgeusia and non-dysgeusia groups were compared using the Kruskal–Wallis test, while categorical variables (sex, ethnicity, education, behavioural factors, and systemic diseases) were assessed using chi-square tests.

For handling missing covariate data, multiple imputation was performed with the missForest algorithm (R package missForest), which leverages random forest models to predict missing values. This non-parametric approach was chosen due to its robustness in handling mixed data types and complex, nonlinear associations among variables.

To estimate the association between dysgeusia and ASCVD, multivariable logistic regression models incorporating NHANES survey weights were constructed, with results reported as odds ratios (ORs) and 95% confidence intervals (CIs))(R package survey::svyglm). We developed three hierarchical models: Model 1 adjusted for age, sex, ethnicity, education level, and BMI; Model 2 included further adjustment for smoking status, alcohol consumption, and HEI score; Model 3 additionally controlled for clinical comorbidities, including hypertension, hyperlipidemia, periodontitis, diabetes mellitus, Parkinson’s disease, and depression. Propensity score matching (PSM) was performed as a sensitivity analysis to minimise confounding (R package MatchIt). Propensity scores were estimated via logistic regression, including age, sex, BMI, race/ethnicity, education, smoking status, alcohol consumption, HEI, hypertension, hyperlipidemia, periodontitis, diabetes, Parkinson’s disease, and depression. A 1:1 nearest-neighbour matching strategy with replacement was applied, using a calliper width of 0.05 standard deviations of the propensity score logit to ensure stringent covariate balance. Post-matching, balance between groups was evaluated using standardised mean differences (SMDs, with an SMD < 0.1 indicating adequate balance) and the Kolmogorov–Smirnov test to confirm equivalence of covariate distributions. Matched samples were then reanalysed with survey-weighted logistic regression to re-evaluate the association between dysgeusia and ASCVD. All analyses were performed using R software (version 4.1), with statistical significance determined by a two-sided P value less than 0.05.

## RESULTS

### Characteristics of the Study Population from NHANES

A total of 6,381 participants met the inclusion criteria, comprising 407 individuals with dysgeusia and 5,974 without. As summarised in Table 1, there were significant differences in demographic characteristics and the prevalence of systemic diseases between the two groups. Compared to participants without dysgeusia, those with dysgeusia were more likely to be female, have a higher BMI, lower HEI scores, and higher rates of ASCVD, diabetes mellitus, hypertension, hyperlipidemia, periodontitis, Parkinson’s disease, and depression.

### Univariate Regression Analysis of Factors Associated with ASCVD

Univariate logistic regression analyses revealed that a range of demographic and clinical variables were significantly associated with ASCVD (Table 2). Older age, male sex, higher BMI, and lower education level were each associated with increased ASCVD prevalence. Several comorbid conditions – including dysgeusia, current smoking, diabetes mellitus, periodontitis, Parkinson’s disease, hyperlipidemia, hypertension, and depression – were also significantly associated with ASCVD, all with P <  0.05.

**Table 2:** Univariate logistic regression analysis of factors associated with ASCVD

Character	Estimate	P	OR	95% CI
Age	0.07	< 0.0001	1.08	1.08 (1.07, 1.09)
Sex				
Female	ref	ref	ref	ref
Male	0.44	< 0.001	1.56	1.56 (1.23, 1.97)
Ethnicity				
mexican	ref	ref	ref	ref
black	0.27	0.07	1.32	1.32 (0.97, 1.78)
white	0.33	0.02	1.39	1.39 (1.06, 1.81)
other	0.1	0.52	1.11	1.11 (0.80, 1.54)
Education				
< 9th grade	ref	ref	ref	ref
9th-12th grade	–0.21	0.11	0.81	0.81 (0.63, 1.05)
>12th grade	–0.77	< 0.0001	0.46	0.46 (0.35, 0.61)
BMI	0.02	0.03	1.02	1.02 (1.00, 1.03)
Smoking				
no	ref	ref	ref	ref
yes	0.66	< 0.0001	1.94	1.94 (1.60, 2.35)
Alcohol consumption				
no	ref	ref	ref	ref
yes	–0.06	0.51	0.94	0.94 (0.78, 1.14)
Diabetes mellitus				
no	ref	ref	ref	ref
PreDM	0.36	0.03	1.44	1.44 (1.03, 2.01)
DM	1.22	< 0.0001	3.4	3.40 (2.74, 4.21)
Periodontitis				
no	ref	ref	ref	ref
mild	0.11	0.87	1.12	1.12 (0.28, 4.51)
moderate	0.96	< 0.0001	2.62	2.62 (2.16, 3.19)
severe	0.57	0.01	1.78	1.78 (1.20, 2.63)
Parkinson’s disease				
no	ref	ref	ref	ref
yes	0.8	0.003	2.24	2.24 (1.34, 3.73)
HEI	0	0.47	1	1.00 (0.99, 1.01)
Hyperlipidemia				
no	ref	ref	ref	ref
yes	1.12	< 0.0001	3.06	3.06 (2.30, 4.07)
Hypertension				
no	ref	ref	ref	ref
yes	1.2	< 0.0001	3.32	3.32 (2.67, 4.13)
Depression				
no	ref	ref	ref	ref
yes	0.85	< 0.0001	2.33	2.33 (1.67, 3.26)
Dysgeusia				
no	ref	ref	ref	ref
yes	0.63	< 0.001	1.88	1.88 (1.39, 2.55)
ASCVD, atherosclerotic cardiovascular disease; BMI, body mass index; HEI, Healthy Eating Index-2015.				

### Multivariable Logistic Regression Analysis of the Association Between Dysgeusia and ASCVD

Multivariable logistic regression analyses demonstrated a positive association between dysgeusia and ASCVD risk. After adjustment for demographic factors (age, sex, ethnicity, education, BMI), lifestyle factors (smoking, alcohol consumption, and HEI), and clinical comorbidities (hypertension, hyperlipidemia, periodontitis, diabetes mellitus, Parkinson’s disease, and depression) in Model 3, dysgeusia remained significantly associated with ASCVD (adjusted OR = 1.46, 95% CI: 1.01–2.13; P = 0.05). These results indicate that participants with dysgeusia had a 46% higher odds of ASCVD even after comprehensive adjustment for potential confounders (Table 3).

**Table 3:** Adjusted odds ratios for the association between dysgeusia and ASCVD across multivariable logistic regression models

ASCVD	Dysgeusia							
	Crude model		Model 1		Model 2		Model 3	
character	95%CI	P	95%CI	P	95%CI	P	95%CI	P
**No**	ref		ref		ref		ref	
**Yes**	1.88 (1.39, 2.55)	< 0.01	1.97 (1.40, 2.76)	< 0.01	1.93 (1.36, 2.73)	< 0.01	1.46 (1.01, 2.13)	0.05
Model 1: Adjusted for BMI, age, sex, ethnicity, and education. Model 2: Additional adjustment for smoking, alcohol consumption and HEI. Model 3: Further adjustment for hypertension, hyperlipidemia, periodontitis, diabetes mellitus, Parkinson’s disease and depression.								

### Propensity Score Matching

To account for potential residual confounding, we conducted propensity score matching (PSM). The propensity scores were estimated using logistic regression that included age, sex, BMI, race/ethnicity, education level, smoking status, alcohol consumption, HEI-2015 score, and clinical comorbidities (hypertension, hyperlipidemia, periodontitis, diabetes mellitus, Parkinson’s disease, and depression). After 1:1 nearest-neighbour matching with a calliper of 0.05 standard deviations, we obtained a balanced cohort of 407 dysgeusia cases and 407 matched controls. In this matched cohort, multivariable logistic regression analysis revealed that dysgeusia was associated with 87% higher odds of ASCVD (adjusted OR = 1.87, 95% CI: 1.08–3.23; P = 0.03) (Table 4). This robust association persisted despite rigorous adjustment through matching.

**Table 4:** Results of multivariable logistic regression analysis for the association between dysgeusia and ASCVD in the propensity score-matched cohort

ASCVD	Dysgeusia							
	Crude model		Model 1		Model 2		Model 3	
character	95%CI	P	95%CI	P	95%CI	P	95%CI	P
**No**	ref		ref		ref		ref	
**Yes**	1.66 (1.04, 2.64)	0.03	1.64 (1.05, 2.56)	0.03	1.64 (1.04, 2.59)	0.03	1.87 (1.08, 3.23)	0.03
Model 1: Adjusted for BMI, age, sex, ethnicity, and education. Model 2: Additional adjustment for smoking, alcohol consumption and HEI. Model 3: Further adjustment for hypertension, hyperlipidemia, periodontitis, diabetes, Parkinson’s disease and depression.								

## DISCUSSION

Alterations in taste perception, collectively termed dysgeusia, have been shown to profoundly influence quality of life, dietary choices, and psychological well-being.^7^ Dysgeusia negatively affects appetite, macronutrient intake, and overall dietary quality, contributing to insufficient nutrition.^10,29^ Notably, recent research has demonstrated that taste dysfunction is strongly associated with increased mortality among acutely hospitalised older adults, underscoring its clinical relevance beyond mere sensory disturbance.^34^

In the present study, our propensity score–matched analysis revealed that individuals with dysgeusia had 87% higher odds of ASCVD compared to matched controls. This finding is consistent with earlier work linking impaired chemosensory function to increased cardiovascular risk, and adds to the emerging evidence that dysgeusia may independently contribute to the development of ASCVD.

Biologically, impaired taste function may influence food preferences, dietary intake, and subsequent disease risk.^22^ The interaction between genetic variation in taste pathways and environmental factors modulates oral sensation and eating behaviour, which in turn shapes cardiometabolic health. Mechanistic studies suggest that taste receptors themselves play direct roles in atherosclerosis pathogenesis. For example, deletion of the taste receptor T1R3 has been shown to reduce atherosclerosis in animal models, an effect largely independent of systemic glucose and lipid levels.^33^ Furthermore, aberrant signalling of cardiac bitter taste receptors (TAS2R family) may promote cardiac inflammation, potentially accelerating atherogenic processes.^41^ Although animal and *in vitro* data suggest that taste receptors may participate in inflammatory signalling relevant to atherosclerosis, human evidence remains limited, and causal inferences should be made cautiously.

Genetic markers of taste function, such as 6-n-propylthiouracil (PROP) bitterness sensitivity and the density of fungiform papillae (FP) on the tongue, have also been implicated as indicators of cardiovascular risk. Individuals classified as nontasters (with low PROP sensitivity) or with reduced FP density are more likely to demonstrate adverse dietary behaviours associated with increased risk of CVD.^8,31^ Genetic and phenotypic markers of taste function may reflect broader sensory and behavioural phenotypes that influence diet and health, but causality has not been established.

### Behavioural Mechanisms: Taste and Eating Behaviour

Dysgeusia substantially alters dietary selection and nutrient intake, potentially worsening cardiometabolic risk profiles. Chronic taste impairment can promote pro-inflammatory dietary patterns, as individuals with reduced taste sensitivity may increase their consumption of sucrose and its derivatives, leading not only to heightened pro-inflammatory cytokine production but also to greater blood glucose fluctuations.^17^ Additionally, diminished taste sensitivity often results in excessive salt use, which has been mechanistically linked to vascular inflammation and subsequent cardiovascular disorders.^6^ Thus, impaired taste function may contribute to the development or progression of ASCVD through adverse changes in eating behaviour.

### Cardiometabolic and Vascular Risk Factors Associated with Dysgeusia

Conversely, several established ASCVD risk factors – including hypertension, obesity, hypercholesterolemia, diabetes mellitus, and chronic kidney disease – have been shown to impair taste function. Large cohort studies in Chinese adults, for instance, have demonstrated that perceived taste dysfunction, as opposed to olfactory impairment, is associated with an increased risk of stroke. Notably, subsequent mediation analyses revealed that chronic diseases such as hypertension, diabetes, chronic kidney disease, and obesity accounted for 4–5% of the association between taste dysfunction and both total and ischaemic stroke, suggesting partial mediation by these cardiometabolic conditions.^39,44^ Furthermore, recent cross-sectional studies have reported that elevated thresholds for salt taste perception in individuals with hypertension are associated with increased CVD risk,^42^ and that higher serum cholesterol concentrations correlate with dysgeusia in Chinese adults.^14^ Poor taste function is also linked to elevated BMI, with a higher prevalence of bitter taste impairment observed in obese individuals.^3^ Additionally, obese subjects exhibit higher detection thresholds across multiple taste modalities – including sweet, salty, bitter, fatty, and sour – and a significantly lower density of fungiform papillae compared to normal-weight controls.^27^ In diabetes mellitus, deficits in flavour and taste recognition are common and may hamper dietary adherence and food selection.^2,25^ Notably, higher glycated haemoglobin (HbA1c) and older age are each associated with decreased taste bud density.^16^ These metabolic and vascular disorders may contribute to dysgeusia through mechanisms involving microvascular compromise in taste bud regions, systemic inflammation, and hormonal dysregulation.^38^

### Inflammation as a Mechanistic Link

Accumulating evidence indicates that systemic inflammation may act as a common mechanistic link between dysgeusia and ASCVD. Both conditions are characterised by elevated levels of pro-inflammatory cytokines and acute-phase reactants such as C-reactive protein, reinforcing the possibility of a bidirectional inflammatory pathway. Type I taste bud cells serve as innate immune sentinels within the oral cavity, exhibiting dynamic immunomodulatory responses to inflammatory stimuli. Recent studies in animal models have shown that macrophage-like properties emerge in these cells, with pronounced upregulation of TNF-α, IL-1β, and IL-6 following exposure to lipopolysaccharide (LPS) or obesity-induced chronic inflammation.^13^ Furthermore, inflammatory insults inhibit taste progenitor cell proliferation and increase cellular turnover, modestly shortening taste cell lifespan and potentially impairing overall taste function.^5^

### Zinc Deficiency: A Biological Intersection

Zinc deficiency represents another biological axis linking dysgeusia and CVD. It is recognised as a cofactor in numerous physiological processes, and inadequate zinc status is associated with elevated blood pressure, dyslipidemia, type 2 diabetes, chronic inflammation, and oxidative stress. Zinc appears to protect against vascular calcification – one pathogenic process in ASCVD – by inhibiting relevant signalling pathways, and higher dietary zinc intake correlates with lower risk of severe arterial calcification.^24^ Zinc is also critical for the structural maintenance of taste cells; deficiency leads to damage of taste bud microvilli and cellular vacuolation, ultimately resulting in diminished taste sensitivity.^12,23^

### Oral Microbiota: Modulator of Taste and Inflammation

The oral microbiota exerts a substantial effect on taste perception and, by extension, dietary preference and metabolic health.^30^ Development of the oral microbial biofilm can physically impede tastant access to their receptors, while microbial metabolism may modify taste sensitivity through the generation or consumption of bioactive metabolites.^26,37^ Furthermore, the emerging concept of the ‘oral-blood axis’ posits that oral bacteria and their inflammatory mediators may disseminate systemically, provoking inflammation in distant organs and contributing to the pathogenesis of diseases such as rheumatoid arthritis and ASCVD.^11^ Thus, alterations in the oral microbial ecosystem may represent a modifiable factor linking dysgeusia to both nutrition and cardiovascular risk.

Importantly, the observed associations between dysgeusia and ASCVD risk factors appear to be bidirectional. Chronic metabolic and vascular diseases can impair taste perception through microvascular injury, inflammation, and hormonal imbalances, while dysgeusia, in turn, may foster unhealthy dietary patterns and exacerbate disease risk. This reciprocal relationship highlights the importance of assessing and addressing chemosensory health in ASCVD risk stratification and prevention strategies.

### Limitations and Prospects

Several limitations should be acknowledged. First, the cross-sectional nature of this study limits the ability to infer causality or determine the temporal sequence between dysgeusia and ASCVD. Second, reliance on self-reported dysgeusia may introduce measurement error due to recall and reporting biases, particularly with respect to the severity and duration of taste dysfunction. Third, although PSM and multivariable adjustments were applied, the possibility of residual confounding from unmeasured factors – such as detailed dietary habits, medication use, subclinical inflammation, and oral health status – cannot be excluded. Future research should prioritise well-designed longitudinal cohort studies to establish the temporal relationship and potential causality between dysgeusia and ASCVD. Additionally, integration of objective assessments of taste function (such as standardised taste tests) and comprehensive metabolomic profiling could help to elucidate the biological pathways linking dysgeusia and ASCVD.

## CONCLUSION

In summary, this study identifies a significant association between dysgeusia and increased odds of ASCVD, independent of established cardiovascular risk factors. These findings highlight the importance of taste function in cardiovascular health and underscore the need for prospective studies to further clarify this relationship and its potential clinical implications.

**Table1:** Baseline population characteristics according to dysgeusia status

Variable	Total	No	Yes	P value
Age				
median (IQR)	56.00 (48.00, 66.00)	56.00 (48.00, 66.00)	56.00 (50.00, 64.00)	0.78
BMI				
median (IQR)	28.30 (24.90, 32.70)	28.20 (24.90, 32.50)	30.20 (25.80, 35.00)	< 0.01
HEI score				
median (IQR)	52.29 (42.96, 62.51)	52.42 (43.11, 62.56)	49.76 (40.61, 60.56)	0.03
Sex, n (%)				< 0.01
female	3312 (52.65)	3052 (51.94)	260 (65.14)	
male	3069 (47.35)	2922 (48.06)	147 (34.86)	
Ethnicity, n (%)				< 0.01
Mexican	682 (5.87)	611 (0.62)	71 (10.32)	
black	1538 (10.39)	1448 (10.30)	90 (11.86)	
white	2738 (72.56)	2590 (73.06)	148 (63.89)	
other	1423 (11.18)	1325 (11.02)	98 (13.94)	
Education, n (%)				< 0.01
< 9th grade	659 (5.60)	588 (5.28)	71 (11.24)	
9th–12th grade	2325 (32.32)	2157 (31.92)	168 (39.34)	
>12th grade	3397 (62.08)	3229 (62.80)	168 (49.42)	
Smoking, n (%)				0.48
no	3364 (52.22)	3160 (52.36)	204 (49.83)	
yes	3017 (47.78)	2814 (47.64)	203 (50.17)	
Alcohol consumption, n (%)				0.05
no	1019 (11.48)	950 (11.29)	69 (14.67)	
yes	5362 (88.52)	5024 (88.71)	338 (85.33)	
Diabetes mellitus, n (%)				< 0.01
no	4163 (71.02)	3946 (71.55)	217 (61.65)	
PreDM	576 (0.24)	537 (9.29)	39 (8.37)	
DM	1642 (19.74)	1491 (19.16)	151 (29.98)	
Periodontitis, n (%)				0.01
no	2716 (54.12)	2556 (54.42)	160 (48.79)	
mild	75 (1.54)	72 (1.60)	3 (0.46)	
moderate	2879 (36.72)	2666 (36.23)	213 (45.31)	
severe	711 (7.63)	680 (7.75)	31 (5.44)	
Parkinson’s disease, n (%)				< 0.01
no	6288 (98.35)	5898 (98.48)	390 (96.13)	
yes	93 (1.65)	76 (1.52)	17 (3.87)	
Hyperlipidemia, n (%)				0.01
no	1488 (22.05)	1415 (22.45)	73 (14.86)	
yes	4893 (77.95)	4559 (77.55)	334 (85.14)	
ASCVD, n (%)				< 0.01
no	5496 (88.08)	5177 (88.51)	319 (80.38)	
yes	885 (11.92)	797 (11.49)	88 (19.62)	
Hypertension, n (%)				< 0.01
no	2792 (48.54)	2665 (49.29)	127 (35.33)	
yes	3589 (51.46)	3309 (50.71)	280 (64.67)	
Depression, n (%)				< 0.01
no	5771 (91.71)	5490 (92.82)	281 (72.15)	
yes	610 (8.29)	484 (7.18)	126 (27.85)	
ASCVD, atherosclerotic cardiovascular disease; BMI, body mass index; HEI, Healthy Eating Index-2015.IQR, interquartile range.				
